# Using commercial dataset for extracting information on nutrition composition for policy evaluation purposes

**DOI:** 10.1017/S1368980025101456

**Published:** 2025-12-23

**Authors:** Beatriz Silva Nunes, Camila Aparecida Borges, Mariana Fagundes Grilo, Ana Clara Duran

**Affiliations:** 1 Graduate Program in Collective Health, School of Medical Sciences, University of Campinas, Campinas, Brazil; 2 Center for Food Studies and Research, University of Campinas (UNICAMP), Campinas, Brazil; 3 Center for Epidemiological Studies in Nutrition and Health, University of São Paulo (USP), São Paulo, Brazil; 4 Department of Food Science and Technology, University of São Paulo (USP), São Paulo, Brazil; 5 Department of Exercise and Nutrition Sciences, The George Washington University, Washington, DC, United States

**Keywords:** Composition nutritional, Food labelling, Nutrition policy, Secondary data analysis, Monitoring, Evaluating

## Abstract

**Objective:**

This study assessed the suitability of nutritional composition data from a commercial dataset for policy evaluation in Brazil.

**Design:**

We compared the proportions of packaged foods and beverages, classified according to the Nova food classification and the nutritional composition of matched products using data from a commercial database of food labels (Mintel-Global New Products Database (GNPD)) and the Brazilian Food Labels Database (BFLD), collected in 2017 as a ‘gold standard.’ We evaluated the agreement between the two datasets using paired *t* tests, Wilcoxon–Mann-Whitney test and the Intraclass Correlation Coefficient (ICC) for energy, carbohydrates, total sugars, proteins, total fats, saturated fats, trans-fats, sodium and fiber.

**Setting:**

Brazil.

**Participants:**

Totally, 11 434 packaged foods and beverages collected in 2017 provided by BFLD and 67 042 packaged foods and beverages launched from 2001 to 2017 provided by Mintel-GNPD.

**Results:**

The proportions of ultra-processed foods (UPF) were similar in both datasets. Paired products exhibited an excellent correlation (ICC > 0·80), with no statistically significant difference in the mean values (*P* ≥ 0·05) of most nutrients analysed. Discrepancies in fibre and fat content were noted in some UPF subcategories, including sweet biscuits, ice cream, candies, dairy beverages, sauces and condiments.

**Conclusion:**

The Mintel-GNPD dataset closely aligns with the BFLD in UPF distribution and shows a similar nutritional composition to a sample of matched foods available for purchase in stores, indicating its potential contribution to monitoring and evaluating food labelling policies in Brazil and in studies of food and beverages composition in food retail through the verification of policy compliance.

To address the rise in non-communicable diseases (NCDs), including obesity, cancer and cardiovascular disease, linked to unhealthy food environments^(1–3)^, World Health Organization (WHO) and Pan American Health Organization (PAHO) have advised governments to develop and implement a set of public policies and regulatory measures to promote healthier food environments^(4,5)^. These measures include restricting the sale of ultra-processed foods (UPF) – industrial formulations containing parts of whole foods and food additives with cosmetic functions, designed to be hyper-palatable and ready-to-consume – in schools, regulating marketing directed at children, taxing sugary drinks, and implementing mandatory front-of-package nutritional labelling (FOPNL)^(2,4–7)^.

Mandatory FOPNL empowers citizens to identify products high in calories, sugars, sodium and fats, while encouraging healthier and informed choices^(8,9)^. Beyond influencing individual purchasing behaviour, the implementation of FOPNL has been shown to drive broader changes in the food environment by encouraging product reformulation^(8–10)^. For instance, a preliminary evaluation of FOPNL’s impact in Chile revealed a significant reduction in the proportion of products labeled as ‘high in’ energy and critical nutrients across multiple food and beverage categories^(11)^. Similarly, in Mexico, within just one year of implementing FOPNL, food manufacturers began reformulating products to lower critical nutrient levels, ensuring compliance with the new labelling standards^(12)^.

In Brazil, nutrition labelling has been mandatory since 2006^(13)^. However, it was not until 2020 that a new food labelling regulation was approved introducing FOPNL alongside other measures, such as restrictions on nutritional claims and enhancements to the nutritional facts label to improve comparability between food products^(14,15)^. Despite these advancements, the limited availability of nutritional composition data for foods in Brazil poses challenges for monitoring and evaluating food labelling policies. This issue is exacerbated by the rapid introduction and updating of new food products, particularly UPF, in the retail market^(16–18)^. To address these challenges, combining commercial and government data is recommended for assessing the retail food market^(16–18)^.

Companies such as Mintel’s Global New Products Database (Mintel-GNPD)^(19)^, Kantar^(20)^ and Euromonitor^(21)^ provide extensive data on food retailing. Each company employs distinct methods to collect this information; for instance, Mintel-GNPD gathers data directly from product labels in various, which may vary across countries for newly launched products on the market^(19)^. Countries including the USA, Chile and Australia have used these commercial datasets to evaluate public policies^(17,22,23)^. However, these datasets may have inherent limitations, and their availability can vary across countries^(24)^.

PAHO recommends evaluating several attributes to select appropriate secondary data for monitoring population health^(25)^. These attributes include population representativeness, ensuring the absence of significant selection bias; periodicity, to enable continuous assessment through regular data collection; validity, ensuring accurate measurement of parameters without distortion, bias or systematic errors; timeliness, emphasising the availability and reliability of data for effective decision-making in the policy process and the potential for data stratification based on specific interests, among other factors^(25)^.

Brazil’s recent nutrition labelling regulations necessitate ongoing monitoring to evaluate their impact on industry compliance, product reformulation and shifts in consumer purchasing behavior. These processes are essential for determining whether the policy’s objectives are being achieved and for resolving unexpected setbacks^(26)^. To date, we have not identified any studies in the literature that evaluate the attributes of commercial databases on Brazilian food and beverage labels, specifically focusing on nutritional composition for monitoring and evaluating policies. However, one study has assessed health and nutrition claims from commercial databases for similar purposes^(27)^. Therefore, this study aimed to assess whether the information on nutrition composition available in this commercial dataset is suitable for policy evaluation in Brazil.

## Methods

### Study design

This cross-sectional study utilised two data sources: secondary data from a private company (referred to as commercial data) and label data collected by Brazilian researchers in supermarkets, which served as the gold standard.

The selected commercial data source fulfills key attributes, such as periodic updates on the Brazilian food supply since 1996; and the timeliness, as it provides current packaged food and beverage label data during the implementation of the new nutrition labelling policy^(25)^. In this study, we focused on two attributes emphasised by PAHO: representativeness and validity. To achieve this, the study used a robust method established in the literature to validate a secondary data source by comparing it with a gold standard or reference dataset^(28,29)^. Using a dataset containing nutrition label information as the gold standard reference, we developed a validation process for the nutritional composition variables in the commercial dataset.

#### Brazilian Food Labels Database sampling procedures

Data from the 2017 Brazilian Food Labels Database (BFLD) served as the ‘gold standard’ due to its standardised sampling, collection, processing and data entry procedures for food and beverage packages. Data collection was conducted by experts in the field, using international nutritional labelling analysis protocols^(30)^. Reliability analyses conducted during data collection ensured data quality, supporting its use in multiple studies across Brazil^(31–33)^. The procedures for data collection and treatment of the primary database will be described below.

Data on food and beverage labels were collected from the five largest food retailers in Brazil – four located in São Paulo and one in Salvador – which together account for 69·7 % of the national grocery retail market share^(31)^. These retailers were selected based on Euromonitor data on annual retail sales volume in Brazil^(31)^. The researchers geo-coded the addresses of all stores belonging to the selected retailers by their companies’ websites and customer service sites. The stores were categorised into low-, middle- or high-income neighbourhoods based on a 1-kilometer Euclidean buffer^(31)^. Neighbourhood income levels were determined using the average per capita family income from the 2010 Brazilian Population Census^(31)^. The stores within each income category were further divided into tertiles based on mean neighbourhood income. From each tertile, one store from the highest and lowest income categories was selected, prioritising stores with larger physical areas. However, for one retail chain, data collection was restricted to its distribution centre. More information can be found elsewhere^(31)^.

Data collection took place between April and June 2017. Fieldworkers photographed all sides of food and beverage packages, excluding alcoholic beverages, nutritional supplements, infant formulas and breast milk substitutes, following the methodology proposed by Kanter *et al.* (2017)^(34)^. Nutritionists entered information from food labels, including nutritional composition, product characteristics, brand and other details, directly into an online platform (Redcap) using previously validated methods^(34)^. Intra- and inter-rater reliability tests were conducted on a 10 % sample of the data using the intraclass correlation coefficient, yielding excellent values (≥ 0·90) for all assessed nutrients^(31)^.

Products entered during the training phase and with duplicate records were excluded, resulting in a total of 12 956 foods and beverage items. Additional exclusions included items with multiple package sizes (*n* 358), multipack products (*n* 86), products lacking nutritional information (*n* 815) or ingredient lists (*n* 178) and those with missing portion sizes and/or calorie values (*n* 85). After these exclusions, the final BFLD comprised 11 434 food and beverage items.

#### Commercial database sampling procedures

For our commercial database sampling, we used data from Mintel, a global private research company that produces the Global New Products Database (GNPD) and other services related to the food consumer experience. This database provides detailed information on packaged food products, including product descriptions, brand, price, package size, claims, nutrition facts and other attributes. Mintel-GNPD continuously monitors the launch of new food products across retailers in more than fifty countries, including Brazil, Chile, Colombia, the United States, Australia, India, China and New Zealand^(19)^. Additional details about Mintel’s data can be found elsewhere^(19)^.

In this study, we analysed data provided by Mintel-GNPD on the nutritional composition of 68 057 unique food and beverage products launched in Brazil between 2001 and 2017. This timeframe was selected because Mintel-GNPD includes newly launched or reformulated products; therefore, by using data from this entire period, we aimed to approximate the prevalence of products available on the market up to 2017. The database includes only the most up-to-date version of each product as of the study’s reference year (2017). We excluded products that do not qualify as foods or beverages under Brazilian food labelling regulations, including dietary supplements (*n* 372), alcoholic beverages (*n* 5) and infant formulas (*n* 79). Additionally, packaged culinary preparations, such as lasagnas or other ready-to-eat frozen meals produced in markets or non-industrial kitchens and commonly available in Brazilian markets, were excluded (*n* 557) as they require a different analytical method and were not the focus of this study. Products without universal product codes (*n* 2) were also excluded, resulting in a final sample of 67 042 food and beverage items.

### Validation process

The validation process was conducted in two stages. First, we analysed the distribution of foods and beverages by categories and subcategories in both databases. Second, we assessed the agreement between products matched across the datasets in relation to the mandatory nutrition facts variables required in Brazil in 2017, including calories, carbohydrates, proteins, total fats, saturated fats, trans-fats, sodium and fibre. Additionally, given the updated nutrition labelling norms published in 2020, which mandated total sugar labelling, we also included total sugar in the analysis.

For the first stage, products in both databases were categorised according to the classification system from the 2017–2018 Brazilian Household Budget Survey, which is based on the Nova food classification. This system includes unprocessed or minimally processed foods (e.g. rice, milk, poultry, beans, beef, fruits, pasta, corn flour, cassava flour, wheat flour, roots and tubers, eggs, vegetables, pork, fish, cereals, offal and other similar foods); processed culinary ingredients (e.g. vegetable oil, sugar, animal fat, starch and other ingredients); processed foods (e.g. bread, cheese, processed meat and other similar items) and UPF, such as cured meats, sweet and savory biscuits, cheese, cakes and pies, bread, candies, carbonated beverages, chocolate, pizza or pastry dough, ready-to-eat meals, non-carbonated beverages, dairy beverages, ice cream, sauces and condiments and other UPF^(35)^.

In addition to these food categories, we introduced a new category called non-sugar sweetener within the ultra-processed subcategories to encompass non-nutritive sweeteners. A detailed description of the foods and beverages included in each category and subcategory is provided in online supplementary material, Supplemental Material 1.

As part of our analysis, we conducted a sensitivity assessment by identifying products in both databases with brands that accounted for up to 80 % of sales in the Brazilian market, as reported by Euromonitor^(21)^. Given that the majority of these brands (approximately 60 %) are classified as UPF, our analysis focused on UPF subcategories^(18)^.

In the second step, we linked the BFLD (11 434 items) and Mintel-GNPD (67 042 items) databases using universal product codes to identify products common to both, enabling the comparison of congruent products to evaluate the agreement between the gold standard database and the commercial database. We excluded products with duplicate universal product codes (*n* 41), resulting in a total of 6065 common food and beverage items across both databases. To ensure the accuracy of paired products, we also verified variables such as product names and descriptions. Details of the foods and beverages from the BFLD that did not match those in the Mintel-GNPD are provided in online supplementary material, Supplemental Material 2.

This new database integrates information from both sources, including product details such as name, description, brand, food categories and nutritional composition. The nutritional data include portion size, energy value, carbohydrates, total sugars, proteins, total fats, saturated fats, trans-fats, sodium and fibre. In the Mintel-GNPD, these data were extracted from a concatenated variable labeled ‘nutrition,’ which combined all the nutritional information. Variables for each nutrient per 100 g or ml were created and subsequently used in the agreement analysis.

### Statistical Analysis

We assessed the distribution of food products through descriptive analysis, applying a 95 % confidence interval (CI), percentage difference (p.p.), and *P* value from proportion test to compare the prevalence of products across both databases. Additionally, we conducted a sensitivity analysis to determine whether, beyond the similarity in the overall distribution of food products, there was alignment in the products from top-selling brands across the databases. To verify this, we repeated the descriptive analyses on products from brands representing up to 80 % of sales in the Brazil^(21)^.

To evaluate the suitability of the Mintel-GNPD’s nutritional composition variables in comparison with a traditionally collected database (BFLD), we conducted paired *t* tests to compare mean values (*P* ≥ 0·05) between matched products in food categories with more than thirty products, and the Wilcoxon–Mann-Whitney test for food categories with fewer than thirty products. The intraclass correlation coefficient (ICC) was also calculated to assess the agreement between matched products, focusing on the total variability of the nutrients of interest^(36)^. For the interpretation of this coefficient with variation from 0 to 1, we used the following parameters: < 0·4 indicates weak correlation; 0·4 to 0·6 reasonable correlation; 0·6 to 0·8 good correlation and > 0·8 excellent correlation^(37)^.

We calculated the agreement between matched products for the following nutritional composition variables: energy (kcal); carbohydrates (g), total sugars (g), proteins (g), total fats (g), saturated fats (g), trans-fats (g), fibre (g) and sodium (mg) per 100 g or ml of foods and beverages. The analysis was performed by food category according to the Nova food classification and for the UPF subcategories, given its higher prevalence among UPF in this study and its significance for public policies^(38)^.

We also evaluated the impact of outliers on energy content on the agreement between matched products as energy information reflects the contribution of other macronutrients (carbohydrates, proteins, and fats). Products with energy values below the second percentile or above the 98th percentile (kcal per 100 g) within each food subcategory were excluded, resulting in a final sample of 5540 products. The agreement analyses, including the paired *t* test or the Wilcoxon–Mann-Whitney test and ICC, were then repeated on this subsample. Furthermore, probability density plots were generated to examine the overlap in the distributions of energy and critical nutrients (total sugar, trans-fat and sodium) across all products in the Mintel-GNPD and BFLD databases.

All statistical analyses were performed using Stata/MP 16.1 (StataCorp LLC) software.

## Results

The BFLD included a total of 11 434 products, with 21·6 % categorized as unprocessed or minimally processed foods, 3·0 % as processed culinary ingredients, 12·7 % as processed foods and 62·7 % as UPF. In comparison, the Mintel-GNPD database contained 67 042 products, with 16·5 % classified as unprocessed or minimally processed foods, 4·3 % as processed culinary ingredients, 15·7 % as processed foods and 63·5 % as UPF (Table 1).

**Table 1.** Percentage of the products in the Brazilian Food Labels Database (BFLD) and Mintel Global New Products Database (Mintel – GNPD), by food categories and subcategories

Food categories	BFLD			Mintel-GNPD			Percentual difference	95 % CI^*^	*P*-value**
	*n*	%	95 % CI^*^	*n*	%	95 % CI^*^			
**Unprocessed or minimally processed foods**	2474	21·6	20·9, 22·4	11 062	16·5	16·2, 16·8	5·1	4·3, 5·9	0·00
Rice	100	0·9	0·7, 1·1	736	1·1	1·0, 1·2	−0·2	–0·4, 0·0	0·03
Milk	23	0·2	0·1, 0·3	166	0·2	0·2, 0·3	0.0	–0·1, 0·0	0·35
Poultry	159	1·4	1·2, 1·6	193	0·3	0·3, 0·3	1·1	0·9, 1·3	0·00
Beans	100	0·9	0·7, 1·1	802	1·2	1·1, 1·3	−0·3	–0·5, –0·1	0·00
Beef	145	1·3	1·1, 1·5	32	0·0	0·0, 0·1	1·3	1·0, 1·4	0·00
Fruits	415	3·6	3·3, 4·0	1357	2·0	1·9, 2·1	1·6	1·2, 2·0	0·00
Pasta	105	0·9	0·8, 1·1	593	0·9	0·8, 1·0	0·0	–0·2, 0·2	0·72
Corn flour and other flours	95	0·8	0·7, 1·0	448	0·7	0·6, 0·7	0·1	0·0, 0·3	0·05
Cassava flour	28	0·2	0·2, 0·4	68	0·1	0·1, 0·1	0·1	0·0, 0·2	0·00
Wheat flour	24	0·2	0·1, 0·3	122	0·2	0·2, 0·2	0.0	–0·1, 0·1	0·52
Roots and tubers	41	0·4	0·3, 0·5	52	0·1	0·1, 0·1	0·3	0·2, 0·4	0·00
Eggs	52	0·5	0·3, 0·6	278	0·4	0·4, 0·5	0·1	–0·1, 0·2	0·54
Vegetables	616	5·4	5, 5·8	1639	2·4	2·3, 2·6	3.0	2·5, 3·4	0·00
Pork	19	0·2	0·1, 0·3	12	0·0	0·0, 0·0	0·2	0·1, 0·2	0·00
Fish	178	1·6	1·3, 1·8	275	0·4	0·4, 0·5	1·1	0·9, 1·4	0·00
Corn, oats and other cereals	59	0·5	0·4, 0·7	274	0·4	0·4, 0·5	0·1	0·0, 0·2	0·10
Offal	28	0·2	0·2, 0·4	9	0·0	0·0, 0·0	0·2	0·1, 0·3	0·00
Other unprocessed or minimally processed foods	287	2·5	2·2, 2·8	4004	6·0	5·8, 6·2	−3·5	–3·8, –3·1	0·00
**Processed culinary ingredients**	346	3·0	2·7, 3·4	2910	4·3	4·2, 4·5	−1·3	–1·7, –1	0·00
Vegetable oils	214	1·9	1·6, 2·1	966	1·4	1·4, 1·5	0·4	0·2, 0·7	0·00
Sugar	62	0·5	0·4, 0·7	398	0·6	0·5, 0·7	−0·1	–0·2, 0·1	0·51
Animal fat	30	0·3	0·2, 0·4	239	0·4	0·3, 0·4	−0·1	–0·2, 0·0	0·11
Starches	21	0·2	0·1, 0·3	234	0·3	0·3, 0·4	−0·1	–0·3, –0·1	0·00
Other processed culinary ingredients	19	0·2	0·1, 0·3	1075	1·6	1·5, 1·7	−1·4	–1·6, –1·3	0·00
**Processed foods**	1450	12·7	12·1, 13·3	10 497	15·7	15·4, 15·9	−3·0	–3·6, –2·3	0·00
Bread	62	0·5	0·4, 0·7	890	1·3	1·2, 1·4	−0·8	–0·9, –0·6	0·00
Cheese	90	0·8	0·6;1·0	385	0·6	0·5, 0·6	0·2	0·0, 0·4	0·01
Processed meats	188	1·6	1·4, 1·9	591	0·9	0·8, 1·0	0·8	0·5, 1·0	0·00
Other processed foods	1110	9·7	9·2, 10·3	8631	12·9	12·6, 13·1	−3·2	–3·8, –2·6	0·00
**Ultra-processed foods**	7164	62·7	61·8, 63·5	42 573	63·5	63·1, 63·9	0·8	–1·8, 0·1	0·08
Cured meats	619	5·4	5·0, 5·8	1369	2·0	1·9, 2·2	3·4	2·9, 3·8	0·00
Sweet biscuits	538	4·7	4·3, 5·1	3606	5·4	5·2, 5·6	−0·7	–1·1, –0·2	0·00
Savory biscuits	469	4·1	3·8, 4·5	3458	5·2	5·0, 5·3	−1·1	–1·5, –0·7	0·00
Cheese	40	0·3	0·2, 0·5	139	0·2	0·2, 0·2	0·2	0·0, 0·3	0·00
Cake and pies	252	2·2	2·0, 2·5	2211	3·3	3·2, 3·4	−1·1	–1·4, –0·8	0·00
Bread	185	1·6	1·4, 1·9	1445	2·2	2, 2·3	−0·5	–0·8, –0·3	0·00
Candies in general	1015	8·9	8·4, 9·4	6926	10·3	10·1, 10·6	−1·5	–2·0, –0·9	0·00
Carbonated sweetened beverages	107	0·9	0·8, 1·1	1240	1·9	1·8, 2·0	−1.0	–1·1, –0·7	0·00
Chocolate	292	2·6	2·3, 2·9	4009	6·0	5·8, 6·2	−3·4	–3·8, –3·1	0·00
Pizza, lasagna or pastry dough	29	0·3	0·2, 0·4	1019	1·5	1·4, 1·6	−1·2	–1·4, –1·1	0·00
Ready-to-eat meals	704	6·2	5·7, 6·6	2276	3·4	3·3, 3·5	2·8	2·3, 3·2	0·00
Non-carbonated sweetened beverages	715	6·3	5·8, 6·7	4810	7·2	7·0, 7·4	−0·9	–1·4, –0·4	0·00
Dairy beverages	610	5·3	4·9, 5·8	2489	3·7	3·6, 3·9	1·6	1·2, 2·1	0·00
Ice cream	288	2·5	2·2, 2·8	1671	2·5	2·4, 2·6	0.0	–0·0, 0·0	0·87
Sauces and condiments	513	4·5	4·1, 4·9	3192	4·8	4·6, 4·9	−0·3	–0·7, 0·1	0·20
Non-sugar sweetener	44	0·4	0·3, 0·5	199	0·3	0·3, 0·3	0·1	0·0, 0·2	0·12
Others ultra-processed foods	744	6·5	6·1, 7·0	2514	3·8	3·6, 3·9	2·7	2·3, 3·2	0·00
Total	11 434	–	–	67 042	–	–	–	–	–

* 95 % CI, **P value from proportion test.

The percentage of UPF showed no statistically significant differences between the BFLD (62·7 %; 95 % CI: 61·8, 63·5) and the Mintel-GNPD (63·5 %; 95 % CI: 63·1, 63·9). Certain UPF subcategories exhibited higher percentages (approximately 1 % variation) in the Mintel-GNPD compared with the BFLD. For example, savory biscuits (BFLD: 4·1 %; Mintel-GNPD: 5·2 %), sweet biscuits (BFLD: 4·7 %; Mintel-GNPD: 5·4 %), cakes and pies (BFLD: 2·2 %; Mintel-GNPD: 3·3 %), bread (BFLD: 1·6 %; Mintel-GNPD: 2·2 %), candies in the general (BFLD: 8·9 %; Mintel-GNPD: 10·3 %), carbonated sweetened beverages (BFLD: 0·9 %; Mintel-GNPD: 1·9 %), non-carbonated sweetened beverages (BFLD: 6·3 %; Mintel-GNPD: 7·2 %) and pizza, lasagna or pastry dough (BFLD: 0·3 %; Mintel-GNPD: 1·5 %). In contrast, the percentage of unprocessed or minimally processed foods was lower in the Mintel-GNPD (16·5 %; 95 % CI: 16·2, 16·8) compared with the BFLD (21·6 %; 95 % CI: 20·9, 22·4) (Table 1).

The sensitivity analyses revealed a higher prevalence of UPF from top-selling brands in the BFLD at 32·1 % (95 % CI: 31·1, 33·2) compared with 25·1 % (95 % CI: 24·7, 25·5) in the Mintel-GNPD (see online supplementary material, Supplemental Material 3). Across most UPF subcategories, the difference in the percentage of products between the two databases did not exceed five percentage points, except for the chocolate subcategory, which exhibited a disparity of 12·8 percentage points.

In the second stage, average nutrient content showed no significant differences between matched products in the two databases across the NOVA food categories (*P* > 0·05), except for carbohydrates per 100 g or ml (*P* = 0·04) (as shown in Table 2). The analysis of UPF subcategories (as presented in Tables 3, 4, and 5) revealed no significant differences (*P* > 0·05) in the average nutrient quantities for cured meats, salted biscuits, cheese, bread, sweetened carbonated beverages, chocolates, ready-to-eat meals, dairy beverages and ice cream between the two databases (BFLD and Mintel-GNPD). However, significant differences were identified in the trans-fat content of sweet biscuits, candies in general, ice cream and the calorie and carbohydrate content of cakes, pies and non-carbonated sweetened beverages.

**Table 2.** Concordance analysis of nutrient amounts per 100 g or ml between matched products from the Brazilian Food Labels Database (BFLD) and Mintel Global New Products Database (Mintel – GNPD) by NOVA food categories

Food categories	Nutrients	*n*	Average of BFLD	Average of Mintel-GNPD	se^*^	Difference of averages	Median of BFLD	Median of Mintel	*P* value**	ICC	CI 95 %***
Unprocessed or minimally processed foods	Energy (kcal)	752	221·3	221·6	1·6	−0·3	205·0	198·0	0·84	0·98	0·98, 0·98
	Carbohydrate (g)	750	34·1	34·2	0·3	−0·2	17·3	16·0	0·64	0·98	0·98, 0·98
	Total sugar (g)	46	11·9	11·8	0·1	0·1	11·8	9·2	0·10	1·00	1·00, 1·00
	Protein (g)	748	9·4	9·5	0·2	−0·1	7·5	7·8	0·47	0·94	0·92, 0·94
	Total fat (g)	656	5·9	5·8	0·1	0·1	1·0	1·4	0·57	0·99	0·98, 0·99
	Saturated fat (g)	738	1·5	1·6	0·0	0·0	0·0	0·0	0·48	0·99	0·99, 0·99
	Trans-fat (g)	732	0·0	0·0	0·0	0·0	0·0	0·0	0·48	0·76	0·68, 0·76
	Fibre (g)	740	5·3	5·3	0·2	−0·1	1·8	1·7	0·81	0·89	0·85, 0·89
	Sodium (mg)	749	43·0	42·7	1·9	0·3	0·3	0·7	0·87	0·95	0·94, 0·95
Processed culinary ingredients	Energy (kcal)	246	691·4	686·9	5·6	4·5	830·8	830·8	0·42	0·97	0·95, 0·97
	Carbohydrate (g)	242	21·6	21·9	0·5	−0·3	0·0	0·0	0·56	0·99	0·99, 0·99
	Protein (g)	243	0·1	0·1	0·0	0·0	0·0	0·0	0·16	0·99	0·99, 0·99
	Total fat (g)	210	77·6	77·8	0·1	−0·2	92·3	92·3	0·17	1·00	1·00, 1·00
	Saturated fat (g)	242	13·9	14·6	0·7	−0·6	14·5	13·8	0·35	0·90	0·84, 0·90
	Trans-fat (g)	242	0·1	0·1	0·0	0·0	0·0	0·0	0·17	0·77	0·63, 0·77
	Fibre (g)	243	0·1	0·2	0·1	−0·1	0·0	0·0	0·33	0·70	0·50, 0·70
	Sodium (mg)	245	29·4	26·7	3·7	2·7	0·0	0·0	0·47	0·96	0·93, 0·96
Processed foods	Energy (kcal)	679	222·0	221·9	4·3	0·1	175·0	176·7	0·98	0·92	0·90, 0·92
	Carbohydrate (g)	672	24·2	24·0	0·4	0·3	11·8	11·1	0·47	0·97	0·96, 0·97
	Total sugar (g)****	27	6·8	6·6	0·2	0·1	5·0	5·3	0·62	1·00	0·99, 1·00
	Protein (g)	674	7·1	6·9	0·1	0·1	4·2	4·2	0·30	0·97	0·96, 0·97
	Total fat (g)	584	12·3	12·1	0·1	0·1	3·7	6·7	0·30	0·99	0·98, 0·99
	Saturated fat (g)	665	3·4	3·4	0·1	0·0	0·6	0·7	0·71	0·97	0·96, 0·97
	Trans-fat (g)	661	0·2	0·2	0·0	0·0	0·0	0·0	0·21	0·96	0·95, 0·96
	Fibre (g)	666	2·3	2·3	0·1	−0·1	1·0	1·2	0·42	0·90	0·86, 0·90
	Sodium (mg)	674	930·3	822·8	58·3	107·5	349·0	343·3	0·07	0·95	0·93, 0·95
Ultra-processed foods	Energy (kcal)	4184	273·1	271·6	1·3	1·4	280·0	277·9	0·26	0·95	0·94, 0·95
	Carbohydrate (g)	4167	36·9	36·4	0·2	0·4	30·0	29·0	0·04	0·95	0·94, 0·95
	Total sugar (g)	578	17·6	17·8	0·2	−0·2	8·5	7·1	0·13	0·99	0·99, 0·99
	Protein (g)	4171	6·3	6·3	0·0	0·0	5·0	5·0	0·83	0·96	0·96, 0·96
	Total fat (g)	3449	13·1	12·9	0·2	0·2	6·5	9·5	0·35	0·87	0·86, 0·87
	Saturated fat (g)	4169	4·6	4·7	0·1	−0·1	2·0	2·1	0·44	0·92	0·91, 0·92
	Trans-fat (g)	4134	0·1	0·2	0·0	0·0	0·0	0·0	0·18	0·73	0·70, 0·73
	Fibre (g)	4148	2·2	2·0	0·2	0·2	0·0	0·0	0·51	0·27	0·18, 0·27
	Sodium (mg)	4175	892·7	899·9	10·7	−7·2	235·5	240·0	0·5	0·99	0·99, 0·99

* SE - Standard Error; **P value of paired *t* test; ***Intraclass Correlation Coefficient (ICC) and CI95% of the ICC; ****Wilcoxon test.

**Table 3.** Concordance analysis of nutrient amounts per 100 g or ml between matched products from the Brazilian Food Labels Database (BFLD) and Mintel Global New Products Database (Mintel – GNPD) by ultra-processed sweet products subcategories

Food categories	Nutrients	*n*	Average of BFLD	Average of Mintel-GNPD	se^*^	Difference of averages	Median of BFLD	Median of Mintel	*P* value**	ICC	CI 95 %***
Sweet biscuits	Energy (kcal)	318	458·0	458·4	6·3	−0·4	450·0	456·7	0·95	0·49	0·37, 0·59
	Carbohydrate (g)	317	66·2	65·5	0·4	0·7	66·7	66·7	0·11	0·82	0·78, 0·86
	Total sugar (g)	50	24·2	24·3	0·2	−0·1	30·5	30·0	0·43	1·00	1·00, 1·00
	Protein (g)	318	6·5	6·9	0·3	−0·4	6·7	6·7	0·16	0·38	0·23, 0·50
	Total fat (g)	318	18·0	18·1	0·1	−0·2	17·6	17·7	0·11	0·98	0·97, 0·98
	Saturated fat (g)	318	7·8	7·8	0·1	0·1	6·3	6·3	0·48	0·95	0·94, 0·96
	Trans-fat (g)	315	0·4	0·6	0·1	−0·2	0·0	0·0	0·00	0·85	0·81, 0·88
	Fibre (g)	318	4·9	3·2	1·2	1·6	2·7	2·7	0·18	0·04	–0·20, 0·23
	Na (mg)	316	245·1	246·0	4·8	−0·9	236·7	236·7	0·85	0·89	0·86, 0·91
Sweet cake and pies	Energy (kcal)	161	365·0	370·1	1·7	−5·1	371·4	373·3	0·00	0·96	0·94, 0·97
	Carbohydrate (g)	160	64·8	64·7	0·6	0·0	60·0	60·0	0·97	0·95	0·93, 0·96
	Total sugar (g)****	6	28·5	28·8	0·3	−0·3	1·7	40·4	1·00	–	–
	Protein (g)	161	5·6	5·1	0·5	0·5	5·2	5·2	0·30	0·14	–0·17;0·37
	Total fat (g)	157	9·6	9·8	0·1	−0·1	8·5	8·7	0·24	0·99	0·99, 0·99
	Saturated fat (g)	161	3·3	4·2	0·8	−0·9	2·6	2·9	0·27	0·25	–0·03;0·45
	Trans-fat (g)	160	0·3	0·3	0·0	−0·1	0·0	0·0	0·01	0·90	0·86, 0·92
	Fibre (g)	161	2·0	2·0	0·1	0·0	1·9	1·8	0·82	0·93	0·90, 0·95
	Sodium (mg)	161	297·0	310·1	4·4	−13·1	276·7	286·7	0·00	0·97	0·95, 0·98
Candies in general	Energy (kcal)	579	345·0	336·3	4·8	8·7	356·0	357·1	0·07	0·76	0·72, 0·8
	Carbohydrate (g)	577	60·8	59·8	0·4	1·1	65·0	65·0	0·01	0·96	0·95, 0·96
	Total sugar (g)	105	28·3	28·3	0·4	0·0	27·0	22·3	0·95	0·99	0·99, 1·00
	Protein (g)	578	5·8	5·9	0·1	−0·1	4·2	4·3	0·48	0·99	0·99, 0·99
	Total fat (g)	435	10·5	10·8	0·1	−0·2	4·6	8·0	0·05	0·99	0·99, 0·99
	Saturated fat (g)	576	4·1	4·2	0·1	−0·1	1·4	1·6	0·02	0·99	0·99, 0·99
	Trans-fat (g)	572	0·1	0·1	0·0	0·0	0·0	0·0	0·03	0·79	0·75, 0·82
	Fibre (g)	572	3·7	2·9	0·7	0·7	0·0	0·0	0·28	0·53	0·44, 0·60
	Sodium (mg)	577	179·2	187·1	4·4	−7·9	80·0	84·0	0·07	0·98	0·98, 0·98
Chocolate	Energy (kcal)	150	504·8	499·6	6·9	5·2	527·3	524·5	0·45	0·80	0·72, 0·85
	Carbohydrate (g)	149	54·9	54·4	0·4	0·5	55·0	55·8	0·20	0·95	0·94, 0·97
	Total sugar (g)	36	34·2	33·9	0·3	0·3	48·0	47·3	0·33	1·00	1·00, 1·00
	Protein (g)	150	6·7	6·7	0·2	0·0	6·4	6·4	0·84	0·90	0·86, 0·93
	Total fat (g)	145	30·2	29·6	0·5	0·6	31·8	32·0	0·24	0·93	0·9, 0·95
	Saturated fat (g)	149	16·3	15·9	0·3	0·4	17·7	17·5	0·10	0·95	0·93, 0·96
	Trans-fat (g)	148	0·2	0·4	0·2	−0·2	0·0	0·0	0·19	0·42	0·20, 0·58
	Fibre (g)	148	3·5	3·8	0·5	−0·3	2·4	2·4	0·55	0·69	0·57, 0·77
	Sodium (mg)	150	88·6	93·9	3·2	−5·3	82·7	83·3	0·10	0·93	0·90, 0·95
Ice cream	Energy (kcal)	157	188·4	194·1	4·3	−5·7	188·3	193·3	0·19	0·83	0·77, 0·88
	Carbohydrate (g)	157	26·4	26·4	0·3	0·0	26·7	26·7	0·98	0·93	0·91, 0·95
	Total sugar (g)****	27	20·2	19·5	0·7	0·8	23·3	23·3	0·28	0·95	0·90, 0·98
	Protein (g)	157	2·8	2·9	0·1	−0·1	2·8	3·3	0·40	0·88	0·83, 0·91
	Total fat (g)	156	8·1	8·1	0·1	0·0	8·3	8·3	0·82	0·98	0·97, 0·98
	Saturated fat (g)	157	4·0	4·1	0·1	−0·1	3·7	3·7	0·51	0·95	0·93, 0·96
	Trans-fat (g)	156	0·1	0·1	0·0	0·1	0·0	0·0	0·03	0·38	0·16, 0·55
	Fibre (g)	157	0·9	0·9	0·0	0·0	0·0	0·0	0·90	0·99	0·98, 0·99
	Sodium (mg)	157	53·7	55·0	1·2	−1·2	52·7	54·0	0·30	0·95	0·93, 0·96
Non-sugar sweetener	Energy (kcal)****	30	188·8	115·3	29·7	73·4	0·0	0·4	0·23	0·72	0·41, 0·87
	Carbohydrate (g)****	29	50·2	26·8	8·4	23·4	0·0	0·1	0·11	0·63	0·21, 0·83
	Protein (g)****	29	0·0	2·1	2·0	−2·1	0·0	0·0	0·50	–	–
	Saturated fat (g)	28	0·0	0·0	0·0	0·0	0·0	0·0	–	–	–
	Trans-fat (g)	28	0·0	0·0	0·0	0·0	0·0	0·0	–	–	–
	Fibre (g)	28	0·0	0·0	0·0	0·0	0·0	0·0	–	–	–
	Sodium (mg)****	28	50·2	122·7	72·3	−72·5	0·0	0·0	0·50	0·59	0·12, 0·81

* SE - Standard Error; **P value of paired *t* test; ***Intraclass Correlation Coefficient (ICC) and CI95% of the ICC; ****Wilcoxon test.

**Table 4.** Concordance analysis of nutrient amounts per 100 g or ml between matched products from the Brazilian Food Labels Database (BFLD) and Mintel Global New Products Database (Mintel – GNPD) by ultra-processed beverages subcategories

Drink categories	Nutrients	*n*	Average of BFLD	Average of Mintel-GNPD	se^*^	Difference of averages	Median of BFLD	Median of Mintel	*P* value**	ICC	CI 95 %***
Carbonated beverages	Energy (kcal)	76	29·8	28·4	1·2	1·4	36·0	35·8	0·25	0·93	0·89, 0·95
	Carbohydrate (g)	76	7·3	6·9	0·3	0·4	9·0	9·0	0·22	0·92	0·87, 0·95
	Total sugar (g)****	28	4·4	4·4	0·0	0·0	5·0	0·3	1·00	1·00	1·00, 1·00
	Protein (g)	76	0·0	0·0	0·0	0·0	0·0	0·0	0·53	0·94	0·90, 0·96
	Total fat (g)	2	1·75	1·50	0·25	0·3	0·0	1·5	–	–	–
	Saturated fat (g)	76	0·0	0·0	0·0	0·0	0·0	0·0	0·32	0·99	0·98, 0·99
	Trans-fat (g)	76	0·0	0·0	0·0	0·0	0·0	0·0	–	–	–
	Fibre (g)	76	0·0	0·0	0·0	0·0	0·0	0·0	0·32	0·97	0·96, 0·98
	Sodium (mg)	76	9·4	9·0	0·4	0·3	7·0	6·1	0·41	0·93	0·89, 0·96
Non-carbonated sweetened beverages	Energy (kcal)	527	121·6	129·5	2·3	−7·9	45·1	47·6	0·00	0·97	0·96, 0·97
	Carbohydrate (g)	525	23·8	25·5	0·5	−1·7	10·5	11·0	0·00	0·97	0·96, 0·97
	Total sugar (g)	166	15·7	16·3	0·4	−0·7	4·5	5·0	0·09	0·99	0·99, 0·99
	Protein (g)	525	1·2	1·2	0·0	0·0	0·0	0·0	0·63	0·99	0·99, 0·99
	Total fat (g)	209	3·3	3·3	0·1	−0·1	0·0	0·3	0·30	0·99	0·99, 0·99
	Saturated fat (g)	526	0·8	0·8	0·0	0·0	0·0	0·0	0·97	0·99	0·98, 0·99
	Trans-fat (g)	524	0·0	0·0	0·0	0·0	0·0	0·0	0·32	0·97	0·96, 0·97
	Fibre (g)	524	0·3	0·3	0·0	0·0	0·0	0·0	0·99	0·94	0·92, 0·95
	Sodium (mg)	528	173·4	180·8	4·4	−7·4	10·3	11·5	0·09	0·98	0·98, 0·99
Dairy beverages	Energy (kcal)	348	93·7	95·1	0·9	−1·5	75·5	77·3	0·09	0·99	0·99, 0·99
	Carbohydrate (g)	347	13·6	13·6	0·2	0·0	12·9	13·0	0·86	0·97	0·96, 0·98
	Total sugar (g)	35	13·0	13·4	0·5	−0·5	8·0	6·5	0·31	0·99	0·98, 1·00
	Protein (g)	347	4·2	4·2	0·1	0·1	3·0	3·0	0·15	0·99	0·99, 0·99
	Total fat (g)	334	2·7	2·7	0·0	0·0	1·8	2·0	0·21	1·00	1·00, 1·00
	Saturated fat (g)	348	1·6	1·6	0·0	0·0	1·0	1·1	0·32	1·00	0·99, 1·00
	Trans-fat (g)	344	0·0	0·0	0·0	0·0	0·0	0·0	0·58	0·99	0·99, 1·00
	Fibre (g)	346	0·2	0·1	0·1	0·1	0·0	0·0	0·25	0·15	–0·05, 0·31
	Sodium (mg)	348	74·1	77·1	2·7	−3·0	57·5	56·1	0·27	0·92	0·90, 0·94

* SE - Standard Error; **P value of paired *t* test; ***Intraclass Correlation Coefficient (ICC) and CI95% of the ICC; ****Wilcoxon test.

**Table 5.** Concordance analysis of nutrient amounts per 100 g or ml between matched products from the Brazilian Food Labels Database (BFLD) and Mintel Global New Products Database (Mintel – GNPD) by ultra-processed other products subcategories

Food categories	Nutrients	*n*	Average of BFLD	Average of Mintel GNPD	se^*^	Difference of averages	Median of BFLD	Median of Mintel	*P* value**	ICC	CI 95 %***
Cured meats	Energy (kcal)	225	226·8	223·3	3·6	3·5	216·0	208·8	0·34	0·92	0·89, 0·94
	Carbohydrate (g)	224	4·9	5·2	0·5	−0·4	2·5	2·3	0·42	0·80	0·74, 0·84
	Total sugar (g)	4	0·50	0·50	0	0	0·5	0·5	–	–	–
	Protein (g)	225	15·9	15·5	0·3	0·4	15·0	15·0	0·13	0·90	0·88, 0·93
	Total fat (g)	225	15·7	15·3	0·3	0·4	15·0	14·9	0·21	0·95	0·93, 0·96
	Saturated fat (g)	224	5·3	5·5	0·2	−0·2	4·5	4·6	0·29	0·82	0·76, 0·86
	Trans-fat (g)	221	0·1	0·1	0·0	−0·1	0·0	0·0	0·15	0·59	0·47, 0·69
	Fibre (g)	222	0·4	0·5	0·1	−0·1	0·0	0·0	0·25	0·94	0·92, 0·95
	Sodium (mg)	223	909·2	898·3	16·5	10·9	733·4	773·3	0·51	0·96	0·95, 0·97
Savory biscuits	Energy (kcal)	313	459·6	450·9	6·8	8·8	450·0	450·0	0·20	0·50	0·37, 0·6
	Carbohydrate (g)	312	60·6	58·3	2·2	2·2	60·0	60·0	0·30	0·36	0·20, 0·49
	Total sugar (g)	52	4·4	4·4	0·1	0·0	4·0	4·3	0·99	0·99	0·99, 1·00
	Protein (g)	311	9·9	9·8	0·1	0·0	8·8	8·8	0·59	0·99	0·99, 0·99
	Total fat (g)	313	19·7	19·7	0·2	0·0	17·6	17·4	0·91	0·98	0·98, 0·99
	Saturated fat (g)	311	5·9	6·1	0·1	−0·2	5·2	5·2	0·07	0·94	0·92, 0·95
	Trans-fat (g)	307	0·3	0·3	0·0	0·0	0·0	0·0	0·92	0·96	0·94, 0·96
	Fibre (g)	310	4·3	4·4	0·2	−0·1	3·7	3·6	0·53	0·81	0·77, 0·85
	Sodium (mg)	311	694·8	729·8	23·0	−35·0	659·3	671·4	0·13	0·73	0·66, 0·78
Cheese	Energy (kcal)	224	283·5	276·3	8·2	7·2	263·3	266·6	0·38	0·71	0·62, 0·77
	Carbohydrate (g)	221	2·9	2·7	0·3	0·2	2·3	2·3	0·35	0·66	0·56, 0·74
	Total Sugar (g)	52	4·4	4·4	0·1	0·0	3·7	3·7	0·99	0·99	0·99, 1·00
	Protein (g)	224	15·3	15·4	0·2	−0·1	12·0	12·0	0·55	0·96	0·95, 0·97
	Total fat (g)	224	22·0	22·2	0·3	−0·2	23·0	23·3	0·52	0·93	0·91, 0·95
	Saturated fat (g)	224	13·4	13·8	0·4	−0·3	13·7	14·5	0·38	0·79	0·72, 0·84
	Trans-fat (g)	222	0·2	0·2	0·0	0·0	0·0	0·0	0·74	0·95	0·93, 0·96
	Fibre (g)	224	2·1	0·1	2·0	2·0	0·0	0·0	0·33	–	–
	Sodium (mg)	224	624·3	625·0	14·7	−0·7	545·0	540·0	0·96	0·88	0·84, 0·90
Bread	Energy (kcal)	109	264·8	267·5	3·4	−2·7	250·0	251·0	0·42	0·88	0·82, 0·92
	Carbohydrate (g)	109	47·1	47·3	0·8	−0·2	46·0	46·0	0·78	0·85	0·78, 0·90
	Total sugar (g)****	11	0·6	0·6	0·1	−0·1	0·0	1·5	1·00	0·98	0·93, 0·99
	Protein (g)	107	9·7	9·3	0·4	0·4	9·4	9·2	0·38	0·68	0·53, 0·78
	Total fat (g)	108	4·5	4·4	0·2	0·1	3·4	3·4	0·53	0·90	0·85, 0·93
	Saturated fat (g)	109	1·1	1·1	0·1	−0·1	0·8	0·8	0·50	0·91	0·87, 0·94
	Trans-fat (g)	107	0·2	0·1	0·0	0·0	0·0	0·0	0·09	0·99	0·99, 0·99
	Fibre (g)	109	4·7	4·7	0·1	0·0	4·6	4·6	0·84	0·98	0·97, 0·99
	Sodium (mg)	109	387·5	395·8	12·9	−8·3	366·0	387·0	0·52	0·72	0·60, 0·81
Ready-to-eat meals	Energy (kcal)	433	285·8	286·8	3·6	−1·0	293·0	291·7	0·78	0·90	0·88, 0·92
	Carbohydrate (g)	431	42·1	42·0	0·3	0·1	40·0	40·0	0·85	0·98	0·98, 0·99
	Total sugar (g)	5	7·0	7·6	0·6	−0·6	4·7	4·4	–	–	–
	Protein (g)	433	8·5	8·6	0·1	−0·1	8·5	8·5	0·05	0·98	0·98, 0·98
	Total fat (g)	429	8·7	8·7	0·1	−0·1	7·0	6·7	0·47	0·98	0·97, 0·98
	Saturated fat (g)	433	3·5	3·5	0·1	0·0	2·5	2·4	0·98	0·95	0·94, 0·96
	Trans-fat (g)	431	0·2	0·2	0·0	0·0	0·0	0·0	0·06	0·91	0·89, 0·93
	Fibre (g)	430	2·4	3·9	1·5	−1·5	1·8	1·9	0·31	–	–
	Sodium (mg)	433	1060·1	1035·0	41·7	25·0	600·0	584·0	0·55	0·88	0·85, 0·90
Sauces and condiments	Energy (kcal)	370	158·5	163·1	2·3	−4·6	133·3	133·3	0·05	0·97	0·97, 0·98
	Carbohydrate (g)	370	16·9	16·7	0·2	0·3	11·0	10·0	0·24	0·98	0·98, 0·98
	Total fugar (g)	39	9·6	10·1	0·3	−0·5	6·2	7·1	0·14	0·99	0·98, 0·99
	Protein (g)	366	2·1	2·0	0·1	0·1	0·0	0·0	0·37	0·96	0·95, 0·97
	Total fat (g)	246	11·3	11·8	0·2	−0·5	0·0	3·9	0·01	0·99	0·99, 0·99
	Saturated fat (g)	365	1·8	1·8	0·1	−0·1	0·0	0·0	0·38	0·95	0·93, 0·96
	Trans-fat (g)	361	0·1	0·0	0·1	0·1	0·0	0·0	0·17	–	
	Fibre (g)	359	0·7	0·7	0·1	−0·1	0·0	0·0	0·09	0·95	0·94, 0·96
	Sodium (mg)	370	5863·3	5911·4	106·7	−48·1	1000·0	958·3	0·65	0·99	0·98, 0·99
Others ultra-processed foods	Energy (kcal)	164	358·4	348·1	6·4	10·3	347·8	345·6	0·11	0·97	0·96, 0·98
	Carbohydrate (g)	163	34·0	31·6	1·2	2·4	17·0	17·3	0·04	0·94	0·92, 0·96
	Total sugar (g)****	12	19·4	19·4	0·0	0·0	21·3	18·3	1·00	1·00	1·00, 1·00
	Protein (g)	164	4·6	4·7	0·1	−0·1	2·4	3·5	0·40	0·99	0·99, 1·00
	Total fat (g)	142	31·6	26·0	4·2	5·6	6·3	7·9	0·18	0·65	0·51, 0·75
	Saturated fat (g)	164	9·7	8·6	1·0	1·1	1·5	1·3	0·28	0·86	0·81, 0·90
	Trans-fat (g)	162	0·4	0·3	0·2	0·2	0·0	0·0	0·49	0·52	0·34, 0·64
	Fibre (g)	164	2·8	2·8	0·1	−0·1	0·0	1·3	0·43	0·99	0·98, 0·99
	Sodium (mg)	164	778·5	771·6	27·5	6·9	166·3	183·3	0·80	1·00	1·00, 1·00

* SE - Standard Error; **P value of paired *t* test; ***Intraclass Correlation Coefficient (ICC) and CI95% of the ICC; ****Wilcoxon test.

The ICC indicated good (ICC: 0·6–0·8) to excellent (ICC > 0·8) agreement for most nutrients across Nova food categories between matched products in the two databases (Table 2). The UPF category demonstrated good to excellent agreement for energy (ICC: 0·95), carbohydrates (ICC: 0·95), total sugars (ICC: 0·99), proteins (ICC: 0·96), total fats (ICC: 0·87), saturated fats (ICC: 0·92), trans-fats (ICC: 0·73) and sodium (ICC: 0·99), but weak agreement for fibre (ICC: 0·27).

Among the UPF subcategories, the ICC results were generally consistent (Tables 3, 4 and 5), showing good to excellent agreement for most nutrients. However, we noted variability in the agreement between databases for specific nutrients, especially in trans-fat and fibre, where the agreement ranged from reasonable (ICC: 0·4–0·6) to weak (ICC < 0·4) in some subcategories, such as cured meats, sweet biscuits, cheese, read-to-eat meals, among others. Both the paired *t* test and ICC did not show improvements in the results when excluding outliers across NOVA classification categories. Despite these discrepancies, probability density plots (see online supplementary material, Supplemental Material 6) indicated that the distributions of energy and critical nutrients were largely overlapping across both databases. This similarity in nutrient distribution supported the predominantly good to excellent agreement observed in the statistical tests conducted on the sample of matched products.

## Discussion

The results indicate similarity in the percentage of UPF between data collected from food and beverage packages in the five major food retailers in Brazil in 2017 and a commercial data provided by Mintel. Furthermore, for most variables (energy, carbohydrates, total sugar, protein, total fat, saturated fat, trans-fat, fibre and sodium), no significant differences were found between matched products, and the agreement across databases for these nutrients ranged from good to excellent. Nevertheless, discrepancies were noted for some nutrients, such as trans-fat and fibre, especially when analyses were further stratified by UPF subcategories.

Researchers in Australia, Spain, India and the UK have used Mintel-GNPD to examine their food and beverages in the food retail and nutritional composition and also found a higher proportion of UPF in their database^(23,39–41)^. As seen in the literature and observed in the present study, this commercial data have the greatest potential for assessing the availability of UPF, rather than unprocessed foods^(42)^. These data demonstrate potential for evaluating UPF composition and supporting regulatory efforts in public health research, especially given the strong association between UPF consumption and health outcomes^(43)^.

In our sample, the Mintel-GNPD includes a smaller proportion of UPF from top-selling brands compared with the BFLD, which can be attributed to the Mintel-GNPD focus on products launched or relaunched in the retail market. However, while this dataset may not encompass all products from top-selling brands available in the Brazilian market, it allows for the monitoring of new market trends and product reformulations, including those from top-selling brands. Additionally, the Mintel-GNPD facilitates the monitoring of the nutritional composition of foods and beverages introduced in the Brazilian retail market, as well as the presence of FOPNL^(44)^, the health and nutritional claims of products^(27)^ and other elements such as advertising and environmental claims.

The nutritional composition information obtained from the Mintel-GNPD aligns with those from the gold standard (BFLD) between matched products for most nutrients across the NOVA food classification categories. However, some discrepancies were observed in the statistical tests for carbohydrates and fibre within the UPF category. For carbohydrates, a *P* value of 0·04 was observed for the difference in means, despite the medians showing minimal variation (BFLD median: 30; Mintel-GNPD median: 29). This discrepancy is likely due to asymmetry in carbohydrate values within this category, characterised by a high concentration of values at zero, common in both zero-calorie and regular products, as illustrated in the Bland–Altman plot in online supplementary material, Supplemental Material 4. For fibre, weak agreement between the datasets was observed, which may be attributed to the ICC’s sensitivity to overall variability rather than just paired variability. In this sample, fibre amounts show variability, with most products reporting values of zero, while some products contain substantial amounts of fibre. This distribution is further illustrated in the Bland–Altman graph provided in online supplementary material, Supplemental Material 5. It is worth noting that the BFLD also has limitations. Unlike the Mintel-GNPD, the BFLD was developed using traditional methods of collecting label information, where researchers followed international protocols for monitoring food labels and had greater control over the data collection process. However, some errors may still be present and may highlight opportunities to improve the data digitisation process by adopting more efficient methods for gathering information from packaged product labels, such as using artificial intelligence.

Within the UPF subcategories, we observed agreement between matched products, although discrepancies were more prevalent for some nutrients, such as trans-fat, fibre and sodium, among others. These discrepancies may be attributed to the size of the sample (due to greater stratification of the data), or specific nuances in nutrition labelling regulations. For example, it is not mandatory to declare trans-fat amounts on labels if the value is equal to or less than 0·1 grams per serving^(14)^. To further investigate this, we randomly reviewed product photographs on the Mintel website and confirmed that the information matched the data recorded in the dataset. Additionally, we verified that the distributions of calories and critical nutrients in both datasets overlapped, confirming the data quality not only for the matched products but also for the entire Mintel-GNPD, as shown in online supplementary material, Supplemental Material 6.

It is important to note that the Mintel-GNPD commercial dataset is not publicly accessible, as its data are produced by the private sector. The specific methods used for data collection and processing are not fully detailed, which may introduce certain limitations. For example, the selection process for data collection of packaged products is unclear. As a result, the dataset may not fully capture the availability of packaged products across different regions, particularly for categories such as unprocessed or minimally processed foods^(45)^. Furthermore, Mintel-GNPD focuses on launched products, which may include items that are not widely consumed in the Brazilian market.

However, commercial packaged food and beverages datasets, such as the one analysed in this study, as well as others such as Simplus^(46)^ and Nielsen^(47)^, are valuable tools for monitoring and evaluating public policies, particularly given the challenges associated with data collection *in loco* ^(48)^. In general, these databases may be expensive; however, they require fewer resources since the data is already collected and structured. Additionally, commercial databases are continuously updated and typically include data from multiple countries, enabling comparisons of food retailing across different regions^(24)^. Nevertheless, it is crucial to assess the quality of this data, as their reliability may vary depending on the location.

This study is not without limitations. The primary limitation stems from the use of universal product codes to match database entries, which can sometimes associate the same UPC with different products. To mitigate this issue, we incorporated additional variables, such as product names and descriptions, into the linking process. Another limitation is that agreement was assessed only for matched products rather than the full Mintel dataset, which may limit generalisability. To address this, we followed validation approaches recommended by the specialised literature, employed a robust gold standard already supporting public policies and analysed a broader sample, in this case, all matched products, representing approximately 53 % of the gold standard dataset. Furthermore, verification of the full dataset was not feasible due to the high cost of data collection and the large volume of products. Nevertheless, probability density plots indicated overlapping distributions of calories and critical nutrients (total sugar, saturated fat, and sodium). A further limitation involves comparing data collected in a single year from the BFLD with accumulated data spanning 2001–2017 from Mintel-GNPD, which may include products no longer available on the market. To address this, we evaluated the suitability of the data for products paired between the two databases. Finally, our study evaluated the sustainability of these commercial data in comparison with Brazilian food retail data, acknowledging potential limitations in extrapolating the findings to products in Mintel’s database from other countries.

However, our results align with a systematic review suggesting that commercial data, particularly purchase and consumption data, can serve as a valuable resource for public health nutrition researchers^(48)^. In addition, to the best of our knowledge, this is the first study to assess the suitability of the Mintel-GNPD dataset in Brazil by comparing it with a food labelling dataset collected and developed by researchers following international protocols for monitoring and evaluating nutrition labelling policies.

## Conclusion

Our findings suggest that Mintel-GNPD provides reliable data on the availability of UPF in the Brazilian retail market and similar nutritional composition to a sample of matched foods available for purchase in stores. The commercial database can support research aimed at monitoring and evaluating labelling and food and beverage composition in Brazil, including policy compliance. Given the opportunities that commercial datasets provide to policy monitoring and impact evaluation, future work should evaluate whether the profile of products in commercial datasets is consistent with foods collected from store shelves in other settings.

## Supporting information

Nunes et al. supplementary material Nunes et al. supplementary material

## Supplementary material

For supplementary material accompanying this paper visit https://doi.org/10.1017/S1368980025101456
