# Supplementary material for: Using commercial dataset for extracting information on nutrition composition for policy evaluation purposes
Source: Public Health Nutr. 2025 Dec 23;28(1):e207. doi: 10.1017/S1368980025101456 (PMC12780808; doi:10.1017/S1368980025101456)

**Supplementary Material**

**Supplementary 1.**

Table 1. Description of the food categories and subcategories.

| **Food category** | **Foods included** |
| --- | --- |
| **Unprocessed or minimally processed foods** |  |
| Rice | Dry rice brown rice. |
| Milk | Whole, semi-skimmed and skimmed milk. |
| Poultry | Poultry including chilled and frozen products. |
| Beans | Dry legumes |
| Beef | Meat including chilled and frozen products. |
| Fruits | Fresh and frozen fruits; frozen fruit pulp |
| Pasta | Dry and chilled pasta |
| Corn flour and other flours | Corn flour and fuba flours. |
| Cassava flour | Cassava flour |
| Wheat flour | Wheat flour |
| Roots and tubers | Potato, cassava, yam. |
| Eggs | Eggs |
| Vegetables | Fresh and frozen vegetables |
| Pork | Pork including chilled and frozen products |
| Fish | Seafood including chilled and frozen products |
| Corn, oats, and other cereals | Dry cereals like corn, oats, quinoa |
| Offal | Meat offal including chilled and frozen products |
| Other unprocessed or minimally processed foods | Teas, coffee, seasonings, nuts, seeds, natural yogurt, other meats. |
| **Processed culinary ingredients** |  |
| Vegetable oils | Vegetable oils and olive oils |
| Sugar | Sugars and honey |
| Animal fat | Butters and fats |
| Starches | Starches and tapioca |
| Other processed culinary ingredients | Salt, baking soda, coconut and fresh cream. |
| **Processed foods** |  |
| Bread | Bread and toasts processed |
| Cheese | Muzarela, cottage, ricota and other types of processed cheese |
| Processed meats | Salted meats, canned fish and smoked meats. |
| Other processed foods | Canned beans and vegetables, sweets in syrup and fruits jam. |
| **Ultraprocessed foods** |  |
| Cured meats | Sausages, seasoned meats, hams, salami, spreads |
| Sweet biscuits | Sweet crackers, biscuits, and cookies |
| Savory biscuits | Savory crackers and appetizers, snacks |
| Margarine | Margarine and vegetable cream |
| Sweet cake and pies | Cakes and pies, including powders |
| Bread | Bread and toast ultra-processed |
| Candies in general | Candies, cocoa powder and sweetened dairy mixes, jellies, syrups, chewing gum, marshmallows, including versions with nonnutritive sweeteners. |
| Carbonated sweetened beverages | Carbonated beverages, including artificially sweetened versions. |
| Chocolate | Products of chocolate, chocolate bars, chocolate spreads |
| Pizza, lasagna, or pastry dough | Pizza, lasagna, or pastry dough |
| Ready-to-eat meals | Ready-to-eat meals, frozen french fries, instant rice, instant noodles, instant soups, instant mashed potatoes, stuffed pasta, frozen pizzas, frozen and ready-to-eat pies, sandwiches, baby foods. |
| Non-carbonated sweetened beverages | Nectars made with juice and added water and/or sugar; fruit drink powder, fruit-flavored beverages; RTD coffee and tea. |
| Dairy beverages | Sweetened yogurt, flavored milks, fermented milk, milk compounds, dairy beverages; unsweetened yogurt, milks, evaporated and powdered milk, milk compounds. |
| Ice cream | Ice cream and sorbets, including versions with nonnutritive sweeteners. |
| Sauces and condiments | Sauces, mayonnaises, herbs, catchup, salad dressings |
| Non-sugar sweetener | Nonnutritive sweeteners |
| Others UPFs | Ultra-processed cheese and breakfast cereals |

**Supplementary 2.**

Table 2. Frequency and percentual (95% confidence interval) of BFLD foods and beverages not matched in Mintel GNPD, categorized by food type.

| **Food categories** | **n** | **% (95% CI*)** |  |
| --- | --- | --- | --- |
|  |  |  |  |
| **Unprocessed or minimally processed foods** | **1629** | **30,9 (29,7; 32,1)** |  |
| Rice | 29 | 0,6 (0,4; 0,8) |  |
| Milk | 7 | 0,1 (0,1; 0,3) |  |
| Poultry | 109 | 2,1 (1,8; 2,5) |  |
| Beans | 32 | 0,6 (0,4; 0,8) |  |
| Beef | 135 | 2,5 (2,1; 3) |  |
| Fruits | 261 | 5 (4,4; 5,6) |  |
| Pasta | 62 | 1,2 (0,9; 1,5) |  |
| Corn flour and other flours | 50 | 0,9 (0,7; 1,2) |  |
| Cassava flour | 13 | 0,2 (0,1; 0,4) |  |
| Wheat flour | 4 | 0,1 (0; 0,2) |  |
| Roots and tubers | 35 | 0,7 (0,5; 0,9) |  |
| Eggs | 30 | 0,6 (0,4; 0,8) |  |
| Vegetables | 526 | 10 (9,2; 10,8) |  |
| Pork | 17 | 0,3 (0,2; 0,5) |  |
| Fish | 127 | 2,4 (2; 2,9) |  |
| Corn, oats, and other cereals | 18 | 0,3 (0,2; 0,5) |  |
| Offal | 26 | 0,5 (0,3; 0,7) |  |
| Other unprocessed or minimally processed foods | 148 | 2,8 (2,4; 3,3) |  |
| **Processed culinary ingredients** | **91** | **1,8 (1,4; 2,1)** |  |
| Vegetable oils | 55 | 1 (0,8; 1,4) |  |
| Sugar | 11 | 0,2 (0,1; 0,4) |  |
| Animal fat | 11 | 0,2 (0,1; 0,4) |  |
| Starches | 6 | 0,1 (0,1; 0,2) |  |
| Other processed culinary ingredients | 8 | 0,1 (0,1; 0,3) |  |
| **Processed foods** | **731** | **14 (13,1; 15)** |  |
| Bread | 41 | 0,8 (0,6; 1,1) |  |
| Cheese | 56 | 1,1 (0,8; 1,4) |  |
| Processed meats | 104 | 2,1 (1,7; 2,5) |  |
| Other processed foods | 530 | 10,1 (9,3; 10,9) |  |
| **Ultra-processed products** | **2800** | **53,4 (52; 54,7)** |  |
| Cured meats | 366 | 6,9 (6,3; 7,7) |  |
| Sweet biscuits | 215 | 4,1 (3,6; 4,6) |  |
| Savory biscuits | 154 | 2,9 (2,5; 3,4) |  |
| Margarine | 14 | 0,3 (0,2; 0,4) |  |
| Cake and pies | 87 | 1,6 (1,3; 2) |  |
| Bread | 73 | 1,4 (1,1; 1,7) |  |
| Candies in general | 417 | 8 (7,3; 8,7) |  |
| Carbonated sweetened beverages | 31 | 0,6 (0,4; 0,8) |  |
| Chocolate | 137 | 2,6 (2,2; 3,1) |  |
| Pizza, lasagna, or pastry dough | 9 | 0,2 (0,1; 0,3) |  |
| Ready-to-eat meals | 248 | 4,7 (4,2; 5,3) |  |
| Non-carbonated sweetened beverages | 180 | 3,4 (3; 3,9) |  |
| Dairy beverages | 213 | 4,2 (3,7; 4,8) |  |
| Ice cream | 130 | 2,4 (2,1; 2,9) |  |
| Sauces and condiments | 132 | 2,5 (2,1; 3) |  |
| Non-sugar sweetner | 7 | 0,1 (0,1; 0,3) |  |
| Others UPPs | 387 | 7,3 (6,7; 8,1) |  |
| Total | 5251 | - |  |

**Supplementary 3.**

Table 3. Percentual and 95% CI of products within the top-selling brands in Brazilian Food Labels (BFLD) and Mintel Global New Products (Mintel - GNPD) databases, by UPFs subcategories.

| **Food subcategories** | **BFLD** | | **Mintel GNDP** | | **Percentual difference** |
| --- | --- | --- | --- | --- | --- |
|  | **n** | **% (95% CI)** | **n** | **% (95% CI)** | **(p.p.)** |
| **Ultra-processed foods** | **2,302** | **32.1 (31.1; 33.2)** | **10,693** | **25.1 (24.7; 25.5)** | **7.0** |
| Cured meats | 208 | 9.0 (7.9; 10.3) | 546 | 5.1 (4.7; 5.5) | 3.9 |
| Sweet biscuits | 214 | 9.3 (8.2; 10.6) | 982 | 9.2 (8.7; 9.7) | 0.1 |
| Savory biscuits | 176 | 7.6 (6.6; 8.8) | 465 | 4.3 (4; 4.8) | 3.3 |
| Margarine | 31 | 1.3 (0.9; 1.9) | 73 | 0.7 (0.5; 0.9) | 0.6 |
| Sweet cake and pies | 74 | 3.2 (2.6; 4) | 585 | 5.5 (5.1; 5.9) | -2.3 |
| Bread | 69 | 3.0 (2.4; 3.8) | 319 | 3 (2.7; 3.3) | 0.0 |
| Candies in general | 240 | 10.4 (9.2; 11.7) | 1,642 | 15.4 (14.7; 16.1) | -5.0 |
| Carbonated beverages | 45 | 2.0 (1.5; 2.6) | 234 | 2.2 (1.9; 2.5) | -0.2 |
| Chocolate | 95 | 4.1 (3.4; 5) | 1,812 | 16.9 (16.2; 17.7) | -12.8 |
| Pizza, lasagna, or pastry dough | 11 | 0.5 (0.3; 0.9) | 217 | 2 (1.8; 2.3) | -1.5 |
| Ready-to-eat meals | 178 | 7.7 (6.7; 8.9) | 485 | 4.5 (4.2; 4.9) | 3.2 |
| Non-carbonated beverages | 281 | 12.2 (10.9; 13.6) | 1,038 | 9.7 (9.2; 10.3) | 2.5 |
| Dairy beverages | 219 | 9.5 (8.4; 10.8) | 707 | 6.6 (6.2; 7.1) | 2.9 |
| Ice cream | 87 | 3.8 (3.1; 4.6) | 428 | 4 (3.6; 4.4) | -0.2 |
| Sauces and condiments | 232 | 10.1 (8.9; 11.4) | 758 | 7.1 (6.6; 7.6) | 3.0 |
| Others UPFs | 142 | 6.2 (5.3; 7.2) | 402 | 3.8 (3.4; 4.1) | 2.4 |

*95% Confidence Interval

**Supplementary 4.**

Figure 1. Bland-Altman plot illustrating the difference in the mean carbohydrates quantity between the Brazilian Food Labels Database (BFLD) and Mintel Global New Products Database (Mintel - GNPD) relative to the mean carbohydrates quantity across both databases within the UPPs category.


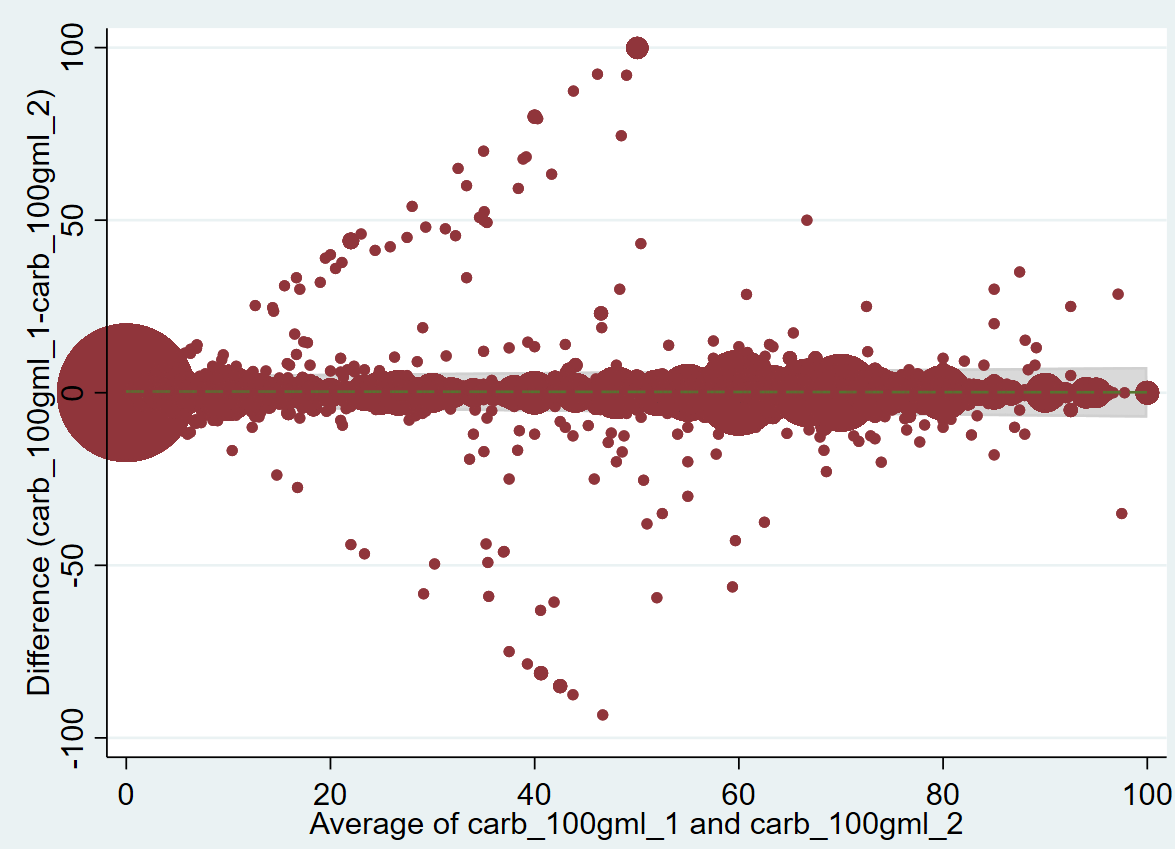


Difference (carbohydrates of BFLD – carbohydrates of Mintel-GNPD)

Average (carbohydrates of BFLD and carbohydrate of Mintel-GNPD)

**Supplementary 5.**

Figure 2. Bland-Altman plot illustrating the difference in the mean fiber quantity between the Brazilian Food Labels Database (BFLD) and Mintel Global New Products Database (Mintel - GNPD) relative to the mean fiber quantity across both databases within the UPPs category.


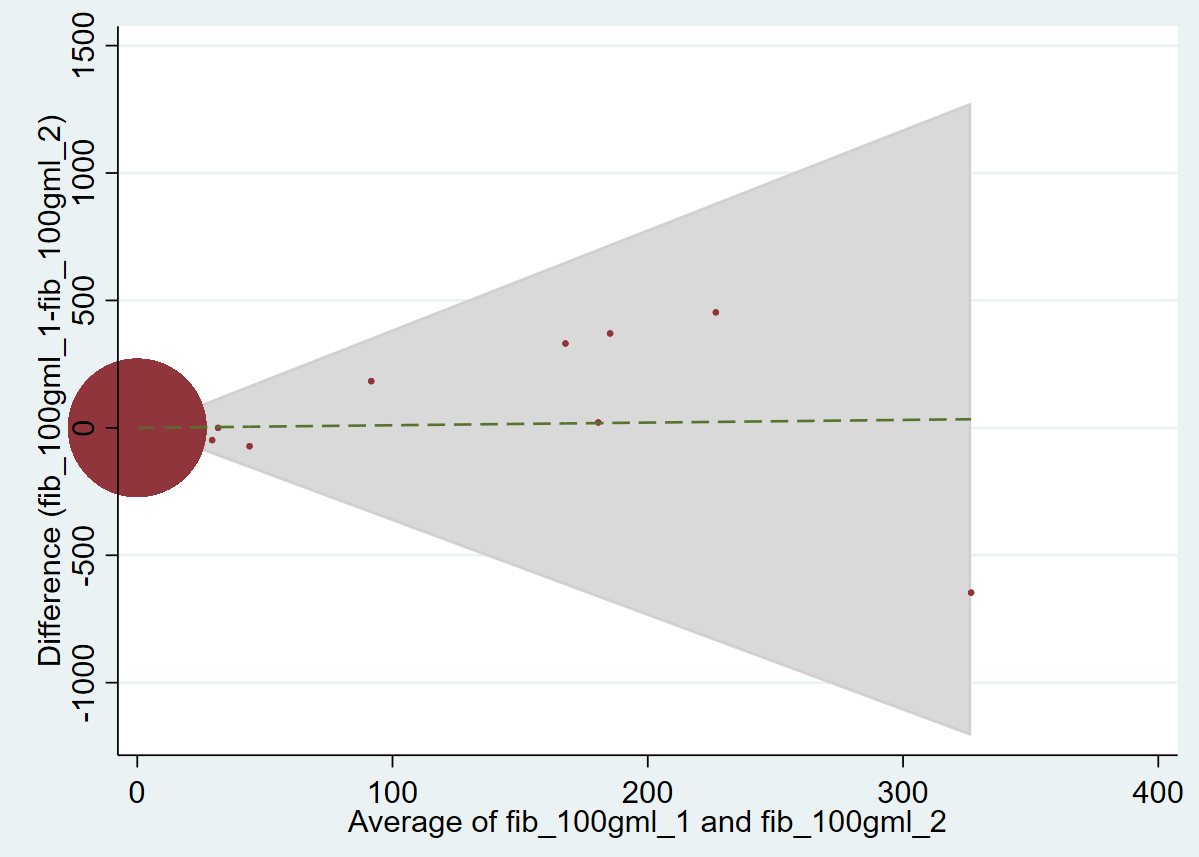


Difference (fiber of BFLD – fiber of Mintel-GNPD)

Average (fiber of BFLD and fiber of Mintel-GNPD)

**Supplementary 6.**6a. Probability density plot of calories for all products from the Brazilian Food Labels Database (BFLD) and the Mintel Global New Products Database (Mintel - GNPD), excluding products with the 99th percentile of calories (n of BFLD: 11,193 and n of Mintel-GNPD: 66,413).


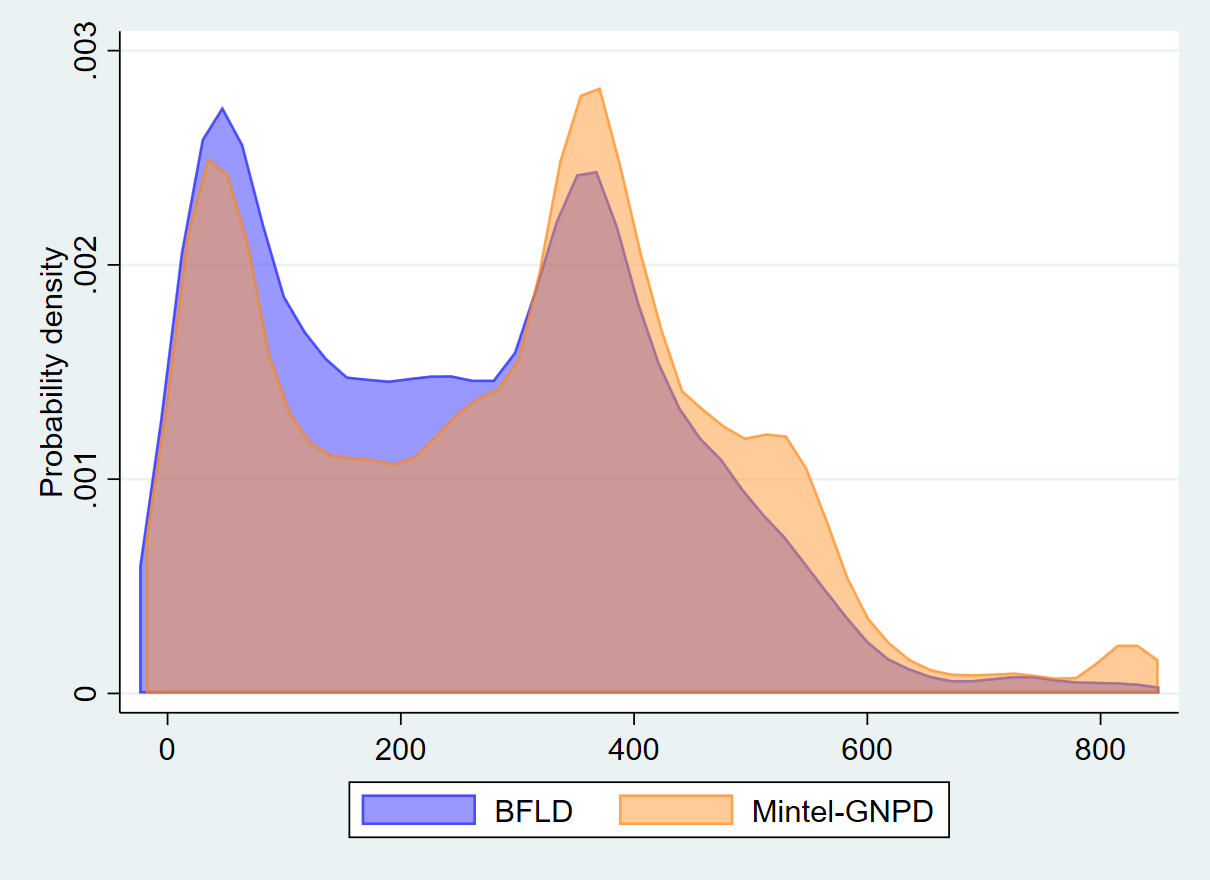


6b. Probability density plot of total sugars for all products from the Brazilian Food Labels Database (BFLD) and the Mintel Global New Products Database (Mintel - GNPD), excluding products with the 99th percentile of total sugars (n of BFLD: 11,419 and n of Mintel-GNPD: 66,995).


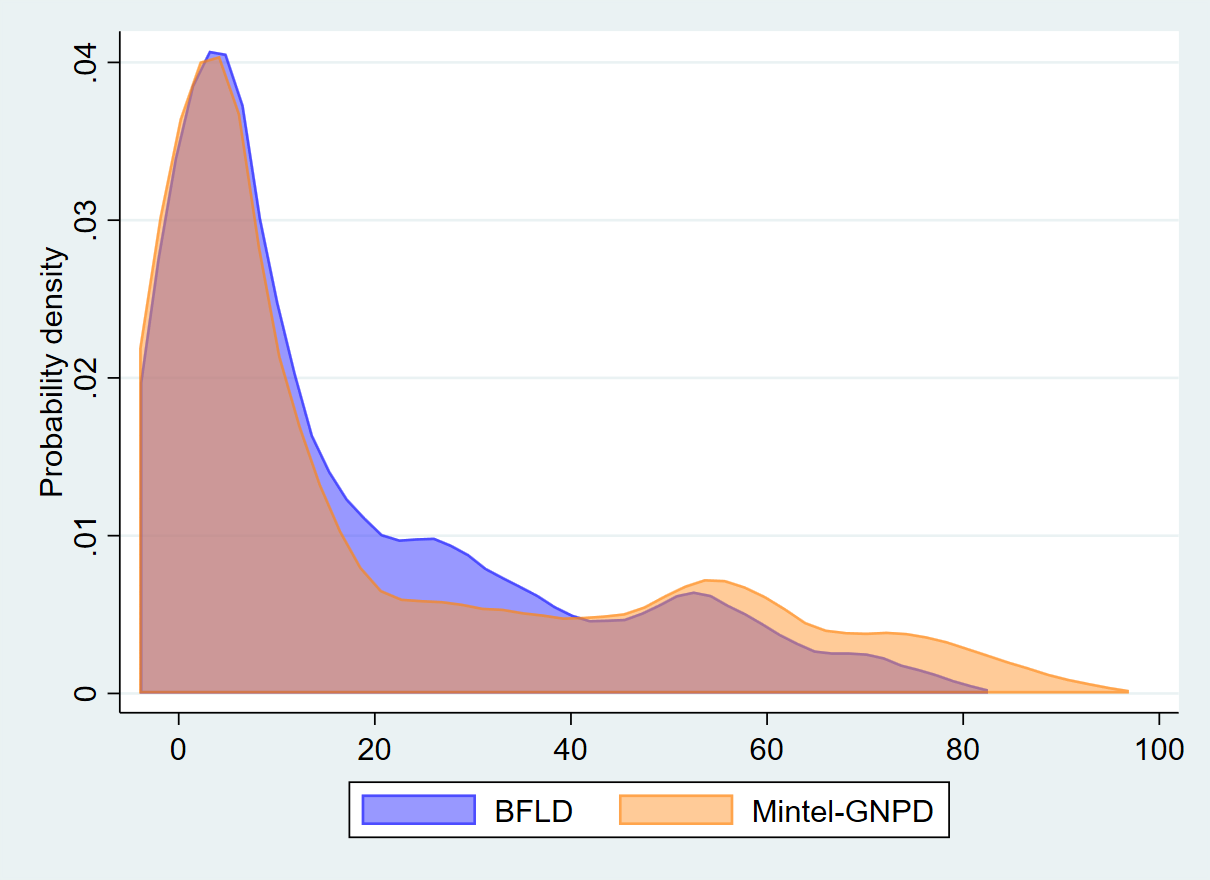


6c. Probability density plot of saturated fat for all products from the Brazilian Food Labels Database (BFLD) and the Mintel Global New Products Database (Mintel - GNPD), excluding products with the 99th percentile of saturated fat (n of BFLD: 11,325 and n of Mintel-GNPD: 66,438).


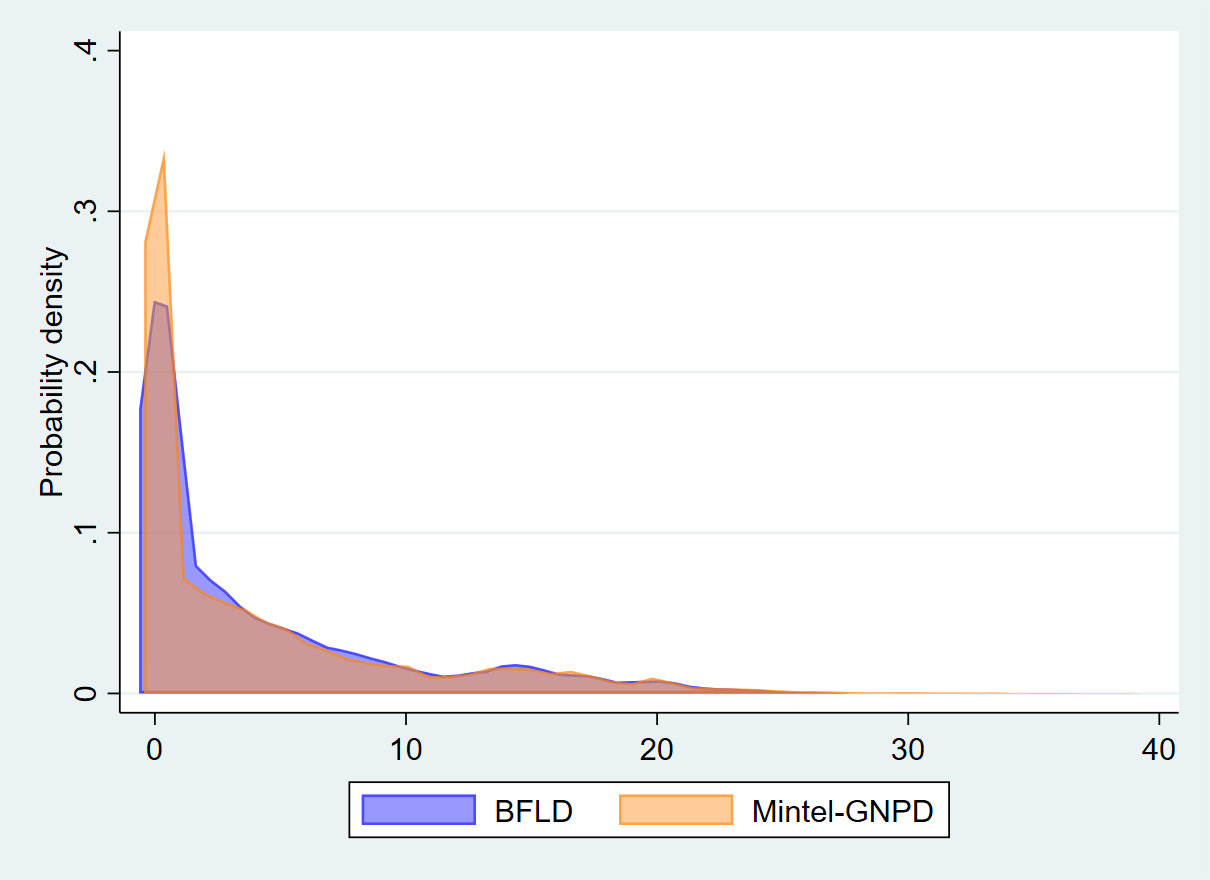


6d. Probability density plot of sodium for all products from the Brazilian Food Labels Database (BFLD) and the Mintel Global New Products Database (Mintel - GNPD), excluding products with the 99th percentile of sodium (n of BFLD: 11,325 and n of Mintel-GNPD: 66,419).


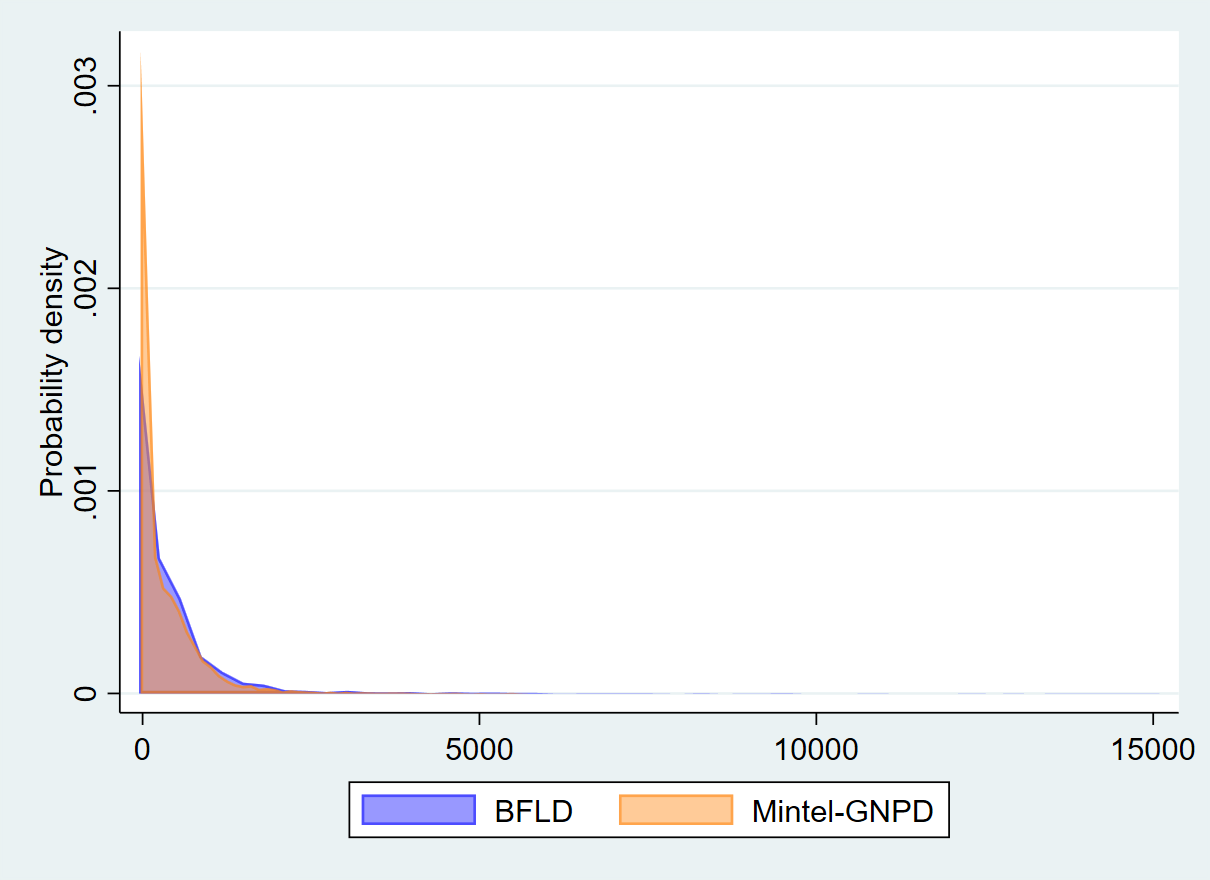

Supplement: Nunes et al. supplementary material [file S1368980025101456sup001.docx]
